# Rotational Thromboelastometry (ROTEM)-Assisted Anaesthetic Management of a Parturient With Paroxysmal Nocturnal Haemoglobinuria and Aplastic Anaemia for Caesarean Section: A Case Report

**DOI:** 10.7759/cureus.95701

**Published:** 2025-10-29

**Authors:** Despoina Fani Papadaki, Emmanouil Stamatakis, Giolanda Varvarousi, Dimitrios Valsamidis

**Affiliations:** 1 Department of Anaesthesiology, Hellenic Red Cross Hospital "Korgialeneio-Benakeio", Athens, GRC; 2 Department of Anaesthesiology, Alexandra General Hospital of Athens, Athens, GRC

**Keywords:** anaesthetic challenges and management, cesarian section, high-risk obstetrics, paroxysmal nocturnal hemoglobinuria (pnh), rotational thromboelastomery (rotem), severe aplastic anemia

## Abstract

Paroxysmal nocturnal haemoglobinuria (PNH) is a rare, acquired, clonal haematopoietic disorder. It is life-threatening and is characterized by intravascular haemolytic anaemia, large vessel thrombosis and bone marrow failure. Aplastic anaemia (AA) and PNH are intimately linked; however, concurrent treatment of PNH and AA is uncommon. Pregnancies in patients with PNH are rare and associated with increased maternal and fetal morbidity and mortality. Nevertheless, with the advent of eculizumab, a monoclonal antibody used to treat PNH, pregnancy outcomes have become more favourable. The obstetric and anaesthetic management of affected patients poses significant challenges, making multidisciplinary involvement essential. We report the successful management of a parturient with PNH and severe AA who underwent a scheduled caesarean section.

## Introduction

Paroxysmal nocturnal haemoglobinuria (PNH) is a rare, nonmalignant, clonal haematopoietic stem cell disease which usually presents with a variety of nonspecific symptoms. Most complications are caused by haemolytic anaemia, thrombosis, or bone marrow failure [1]. It typically occurs in early adulthood, while children are rarely affected by the disease. The exact epidemiology of PNH is unknown, although the prevalence of the disease is estimated to be at least 15.9 per million individuals, with an incidence of 1-1.5 cases per million people per year [1,2]. Epidemiologic data reported in the literature can vary significantly across countries, with different complications occurring more frequently in different countries [1,2]. Despite the condition being attributed to an X-linked chromosome mutation, the disease affects women slightly more frequently than men [1].

PNH is caused by somatic mutations in one or more clones of the haematopoietic stem cells, resulting in a complete or partial deficiency in glycosylphosphatidylinositol (GPI)-anchored proteins. Such proteins include CD55 and CD59, which act as complement inhibitors and play a role in protecting red blood cells from complement-mediated lysis. When a mutated clone acquires a growth advantage and differentiates, mature blood cells lacking these protective proteins are generated, leading to a chronic complement-mediated haemolysis. This process can be exacerbated by further complement activation due to stress caused by surgery, trauma, pregnancy, inflammation, or other triggers [1,2].

In the past, PNH treatment was mostly supportive, with a 10-year survival rate of only 50%. The introduction of complement inhibitors revolutionised PNH management, significantly improving survival to near-normal levels. These include eculizumab and ravulizumab, two monoclonal antibodies widely used for PNH treatment. Eculizumab has long been the standard therapy for PNH and remains the most widely used complement inhibitor. Ravulizumab, a newer monoclonal antibody, offers several advantages over eculizumab, including extended dosing intervals that reduce treatment burden. However, long-term data on its safety and efficacy are still limited [1]. Allogeneic haematopoietic stem cell transplantation remains the only curative therapy for PNH. However, it is rarely performed due to the substantial risks of morbidity and mortality, the small chance of spontaneous remission (approximately 2%), and the proven efficacy of eculizumab in controlling the disease. It is therefore generally reserved for patients with severe bone marrow failure [2]. Supportive therapies, including transfusions of blood products, anticoagulation, as well as iron, folic acid and vitamin B12 supplements, continue to play a significant role in treating symptoms and complications of the disease [1,2].

The overlap between idiopathic aplastic anaemia (AA) and PNH has been extensively documented. Each condition can develop into another or occur simultaneously. AA is a bone marrow failure caused by the attack of autoreactive cytotoxic T lymphocytes in the haematopoietic stem cell compartment [3]. Patients with AA often have PNH clones; however, only a few develop clinically evident PNH. Respectively, patients with PNH typically have some kind of bone marrow failure, yet only a few require concurrent treatment for AA. Currently, aplastic PNH is considered one of the three subtypes of PNH. The prognosis of PNH patients with a profound bone marrow failure is typically worse compared to patients with the haemolytic type of PNH receiving treatment [2,3]. The concurrent treatment of AA and PNH presents unique therapeutic challenges, as standardised treatment recommendations are lacking. Therefore, management strategies are informed primarily by case reports and small case series [3-5].

Limited information exists on the prevalence of PNH in pregnant women. Both PNH and AA are associated with adverse maternal and fetal outcomes. Before the introduction of eculizumab, pregnancy was discouraged due to the high risks of maternal and fetal morbidity and mortality [6]. Although studies on the effects of eculizumab are limited to retrospective designs and subject to patient selection bias, pregnancy outcomes appear to have improved with its use [6]. Nonetheless, significant complications persist, including thrombosis, infections, haemorrhage, anaemia, infant death, prematurity, and spontaneous abortion. Reported maternal and perinatal mortality rates remain substantial, at 10-20% and 5-10%, respectively [2,7].

The coexistence of PNH and AA in pregnancy presents a unique and demanding challenge for anaesthesiologists, who must carefully manage multiple, sometimes contradictory risks, such as profound bleeding due to pancytopaenia and a high thrombotic potential inherent to PNH. Anaesthesiologists must anticipate rapid changes in haemodynamic and haemostatic status, optimise transfusion strategies, and coordinate closely with obstetricians and haematologists to ensure maternal and fetal safety. Rotational thromboelastometry (ROTEM) provides a dynamic, real-time assessment of haemostasis by measuring the viscoelastic properties of blood during clot formation and lysis. It offers detailed information on clot initiation, formation, and stability, complementing conventional coagulation tests. It may help guide transfusion and coagulation management, although further research is needed to establish its role in this setting. Reports of anaesthetic management in pregnant patients with concurrent PNH and AA are exceedingly rare, making this case a valuable contribution to the literature. This report underscores the importance of multidisciplinary planning and suggests that point-of-care coagulation monitoring could be beneficial in these high-risk patients, although further research is needed to evaluate this potential benefit.

## Case presentation

We present the case of a 25-year-old woman, gravida 1 (G1), para 0 (P0) with AA-PNH. She was diagnosed in the fifth gestational week following a pancytopaenia workup. She had no prior medical history. Treatment with eculizumab and cyclosporine was initiated shortly after, the latter administered for the management of severe AA. After receiving 600 mg of eculizumab weekly for four weeks as an induction dose, she continued maintenance therapy with 900 mg every 14 days throughout her pregnancy. Cyclosporine levels were also monitored according to haematology protocols. She also received iron and folic acid supplementation throughout the pregnancy. Despite treatment, the patient had persistent AA, requiring at least weekly transfusions of platelets and erythrocytes. Later in gestation, transfusions, particularly platelets, were required almost daily. She remained hospitalised from her 28th week of pregnancy and was scheduled for a bone marrow transplant following parturition. At 29 weeks of gestation, she developed severe neutropaenia and neutropaenic fever, with a white blood cell count of 0.5 x 10 K/mcl and a neutrophil count of 0.2 x 10 K/mcl. Apart from the antibiotic treatment, filgrastim 30 mcg daily was initiated after haematology consultation. The neutropenic fever gradually subsided, while her neutrophil counts increased to near-normal levels.

At 33 weeks and three days of gestation, a scheduled caesarean section was performed. The preoperative complete blood count showed a haematocrit of 25.8%, haemoglobin of 9 g/dl, platelet count 3 x 10 K/mcl, white blood cell count of 4 x 10 K/mcl, while coagulation testing was within normal limits. Biochemical tests showed normal kidney and renal function, and lactate dehydrogenase (LDH) was 178 U/L (Table 1). Following a haematology consultation, she received a transfusion of three units of platelets (each approximately 50 mL) 30 minutes prior to arriving in the operating room, in order to achieve the optimal platelet levels for the caesarean section. An additional five units of red blood cells (approximately 250 ml each) and three units of platelets (approximately 50 ml each) were also secured from the blood bank in advance. Her last dose of eculizumab had been administered six days before surgery, in accordance with her regular 14-day treatment schedule.

**Table 1:** Laboratory results preoperatively * indicates values outside of the normal reference range

Parameter	Normal range	Results
White blood cells (K/μL)	4.5-10.5	4.0*
Neutrophils (%)	40.0-70.0	22.0*
Lymphocytes (%)	20.0-45.0	61.6*
Monocytes (%)	2.0-12.0	16.1*
Eosinophils (%)	0.0-6.0	0.2
Basophils (%)	0.0-2.0	0.1
Neutrophils (K/μL)	1.8-7.0	0.9*
Lymphocytes (K/μL)	1.2-3.8	2.5
Monocytes (K/μL)	0.2-1.0	0.6
Eosinophils (K/μL)	0.0-0.6	0.0
Basophils (K/μL)	0.0-0.2	0.0
Red blood cells (M/μL)	4.5-6.00	2.95*
Haemoglobin (Hgb, g/dL)	14.0-17.5	9.0*
Haematocrit (Hct %)	42.0-52.0	25.8*
Mean corpuscular volume (MCV, fL)	79.0-98.0	87
Mean corpuscular haemoglobin (MCH, pg)	26.0-32.0	30.5
Mean corpuscular haemoglobin concentration (MCHC, g/dL)	32.0-36.0	34.9
Red blood cell distribution width-coefficient of variation (RDW-CV, %)	11.5-14.9	14.6
Platelets (K/μL)	140-440	3.0*
Mean platelet volume (MPV, fL)	7.0-10.5	10.3
Plateletcrit (%)	0.140-0.420	0.003*
Platelet distribution width-coefficient of variation (PDW, %)	12.0-18.0	16.9
Potassium (K, mmol/L)	3.5-5.1	4.2
Sodium (Na, mmol/L)	136-145	138
Alanine transaminase (ALT, U/L)	<41	7
Aspartate transaminase (AST, U/L)	<37	12
Urea (mg/dL)	6-22	19
Glucose (mg/dL)	65-110	89
Creatinine (mg/dL)	0.6-1.2	0.37
Gamma-glutamyl transferase (GGT, U/L)	11-49	15
Uric acid (mg/dL)	<5.7	4.4
Total bilirubin (mg/dL)	0.2-1.2	0.21
Total proteins (g/dL)	6.4-8.3	5.5*
Albumin (g/dL)	3.5-5.0	3.4*
C-reactive protein-high sensitive (mg/dL)	<0.4	0.99*
Lactate dehydrogenase (LDH, U/L)	135-225	178
Prothrombin time (PT, sec)	11.0-15.0	9.8*
International normalised ratio (INR)	-	0.92
Activated partial thromboplastine time (APTT, sec)	26.0-40.0	19.7*
Fibrinogen (g/L)	2.0-4.0	3.4

During the pre-anaesthetic assessment, airway evaluation revealed a Mallampati score of II, a thyromental distance greater than 6 cm and a normal interincisal distance. Given the markedly reduced platelet count, general anaesthesia was selected, and informed consent was obtained from the patient.

**Figure 1:**
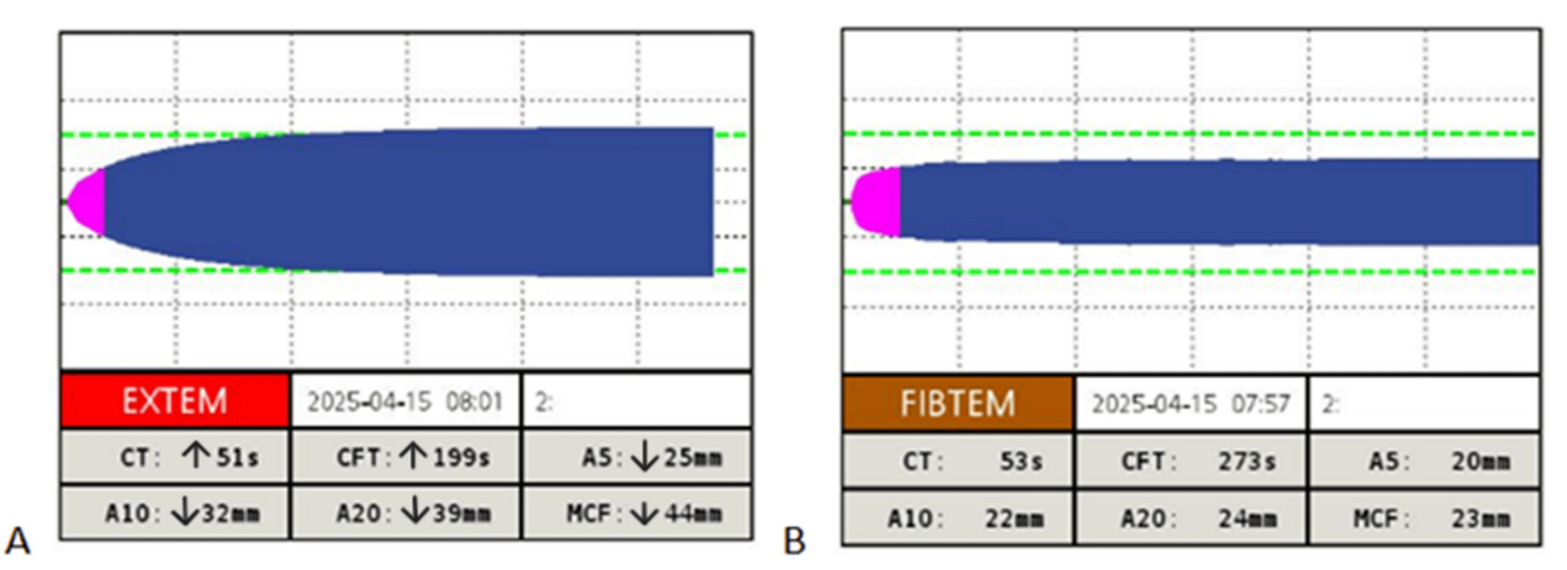
EXTEM and FIBTEM results upon arrival in the operating room (preoperatively) EXTEM: extrinsically activated assay with tissue factor; FIBTEM: fibrinogen-based thromboelastometry Clotting time (CT): time from test initiation until the first detectable clot formation, primarily reflecting coagulation factor activity. Clot formation time (CFT​): time from the beginning of clot formation until a defined firmness is reached, reflecting the rate of fibrin polymerisation and platelet interaction. A5, A10, A20 (amplitude at 5, 10, and 20 minutes): these parameters indicate clot firmness at successive time points and provide early information on clot kinetics. Maximum clot firmness (MCF): the peak amplitude reached during the test, representing the final clot strength, which depends on both platelet function and fibrinogen concentration. The values displayed at the bottom of each panel represent the patient’s results, with arrows indicating whether they fall above or below the normal reference ranges. Normal reference ranges according to the manufacturing company: EXTEM: CT, 38-79 s; CFT, 34-159 s; A10, 43-65 mm; A20, 50-71 mm; MCF, 50-72 mm. FIBTEM: CT not determined, CFT not determined, A10 7–23 mm, A20 8–24 mm, MCF 9–25 mm. (A) EXTEM tracing (left): The patient’s results demonstrate a prolonged CFT (199 s) and reduced A5 (25 mm), A10 (32 mm), A20 (39 mm), and MCF (44 mm), consistent with delayed and weak clot formation, likely secondary to severe thrombocytopenia. Although the manufacturer does not provide formal reference ranges for A5, it is clinically useful as an early indicator of clot firmness and correlates well with A10 and MCF, allowing timely therapeutic decisions in rapidly changing clinical settings. (B) FIBTEM tracing (right): Values are within normal limits, indicating adequate fibrinogen contribution to clot formation despite marked thrombocytopaenia

In the operating room, two peripheral intravenous lines (18G and 16G) were placed. Standard monitoring was applied, and an arterial line was inserted. A rotational thromboelastometry (ROTEM Tem Innovations GmbH, Munich, Germany) sample was obtained prior to the caesarean section. It revealed prolonged EXTEM clot formation time (CFT 199 s) and reduced EXTEM A5 (25 mm), A10 (32 mm), and MCF (44 mm), consistent with delayed and weak clot formation due to thrombocytopenia (Figure 1A). Fibrinogen-based thromboelastometry (FIBTEM) values were within normal limits (Figure 1B). Based on these findings, an additional unit of platelets was transfused. Anaesthesia induction was achieved with propofol (2.5 mg/kg) and rocuronium (0.9 mg/kg) and was uneventful.

**Figure 2:**
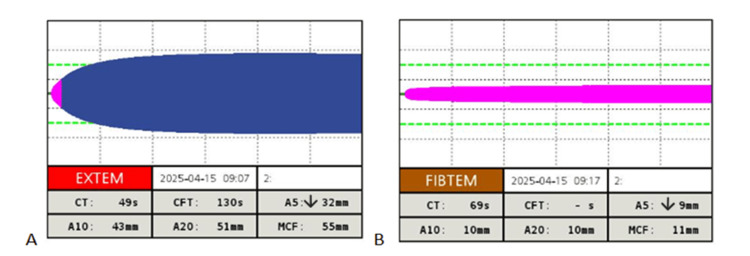
EXTEM and FIBTEM results intraoperatively following platelets transfusion EXTEM: extrinsically activated assay with tissue factor; FIBTEM: fibrinogen-based thromboelastometry; ROTEM: rotational thromboelastometry ROTEM parameters are described in detail in Figure 1. The values displayed at the bottom of each panel represent the patient’s results, with arrows indicating whether they fall above or below the normal reference ranges. Normal reference ranges according to the manufacturing company: EXTEM: CT, 38-79 s; CFT, 34-159 s; A10, 43-65 mm; A20, 50-71 mm; MCF, 50-72 mm. FIBTEM: CT, not determined; CFT, not determined. A10 7-23 mm, A20 8-24 mm, MCF 9-25 mm. (A) EXTEM tracing demonstrates normal CFT, but reduced early clot firmness (A5) and borderline A10 and A20 values, suggesting compromised clot stability and impaired platelet contribution to clot formation. Although formal reference ranges for A5 are not provided by the manufacturer, it remains clinically valuable as an early indicator of clot firmness, correlating closely with A10 and MCF and aiding timely therapeutic decisions. (B) FIBTEM tracing demonstrating reduced A5 values, with A10 and A20 near the lower limit of normal, indicating impaired fibrinogen contribution to clot formation

Intraoperatively, arterial blood gases (ABG) analysis was performed (Table 2). Due to low haemoglobin levels, as well as ongoing surgical bleeding, one unit of red blood cells was administered. Calcium chloride 10% was also given as indicated. Following transfusion of the additional unit of platelets and the intraoperative unit of red blood cells, a full blood count, an anticoagulation profile and another ROTEM sample were obtained. The second ROTEM demonstrated low EXTEM A5 (32 mm), improved A10 and A20, and normal CFT values (Figure 2A). In addition, FIBTEM showed reduced A5 (9 mm) with A10 and A20 at the lower limit of normal, indicating impaired fibrinogen contribution to clot formation (Figure 2B). Given the patient’s high thrombotic risk, only 1 g of tranexamic acid was administered. Further management, including the use of fibrinogen concentrate, was deferred until reassessment. After communication with the obstetricians, 100 mcg of carbetocin and 200 mcg of ergometrine were given intravenously to avoid postpartum haemorrhage caused by uterine atony in this high-risk patient. A male neonate was delivered (2375 g) with an Apgar score of 8 and 9 at the first and fifth minutes, respectively. Following the delivery, 250 mcg of fentanyl, 8 mg of morphine, 1 g of paracetamol, and 50 mg of dexketoprofen were administered for analgaesia. Estimated intraoperative blood loss was approximately 1.1 L, with 1.6 L of intravenous fluids administered and urine output maintained at 2 mL/kg/h. The patient remained haemodynamically stable throughout the procedure and was extubated uneventfully. The total duration of surgery was 65 minutes, while anaesthesia lasted 75 minutes.

**Table 2:** Intraoperative arterial blood gas analysis Hgb: haemoglobin; Hct: haematocrit; HCO3: bicarbonate; SO_2_: oxygen saturation; pCO_2_: partial pressure of carbon dioxide; pO2: partial pressure of oxygen Ventilator settings: tidal volume, 450 ml; respiratory rate, 14 breaths per minute; positive end-expiratory pressure (PEEP), 5; FiO_2_, 35%

Parameter	Result
pH	7.45
pCO_2_	33
pO_2_	104
Na^+^	136
K^+^	3.7
Ca^++^	0.74
Glucose	91
Lactate	2.4
Hgb	8.2
Hct	26.5
HCO_3_	22.9
Base excess	-1.1
SO_2_	100%

**Figure 3:**
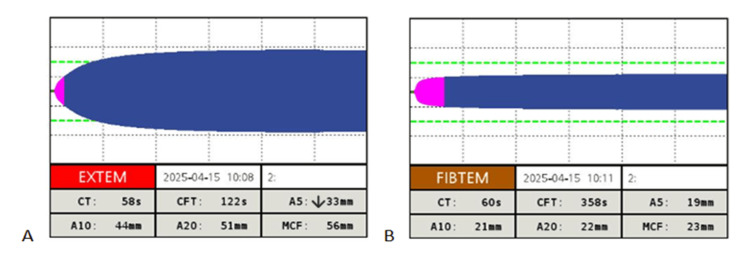
EXTEM and FIBTEM results following 1 g of tranexamic acid administration (postoperatively) EXTEM: extrinsically activated assay with tissue factor; FIBTEM: fibrinogen-based thromboelastometry; ROTEM: rotational thromboelastometry ROTEM parameters are described in detail in Figure 1. The values displayed at the bottom of each panel represent the patient’s results. Normal reference ranges according to the manufacturing company: EXTEM: CT, 38-79 s; CFT, 34-159 s; A10, 43-65 mm; A20, 50-71 mm; MCF, 50-72 mm; FIBTEM: CT, not determined; CFT, not determined; A10, 7-23 mm; A20, 8-24 mm; MCF, 9-25 mm (A) EXTEM tracing revealing a slightly low A5 value, with A10 and A20 values at the lower end of the normal values, indicating partial recovery of clot formation. While A5 lacks manufacturer-defined reference ranges, it serves as a practical early marker of clot firmness, showing good correlation with A10 and MCF, and is frequently used to guide rapid perioperative decision-making. (B) FIBTEM tracing with all values within the normal reference range

While the patient was in the recovery room, the results from intraoperative laboratory testing became available, showing a haematocrit of 26.4%, haemoglobin of 9.3 g/dl, a platelet count of 52 × 10³/µL, and a normal coagulation profile (Table 3). At the same time, final ABG and ROTEM samples were obtained. The ABG analysis demonstrated a haematocrit of 28% and haemoglobin of 9 g/dl (Table 4), while ROTEM revealed a slightly low EXTEM A5 value of 33 mm, with all other EXTEM and FIBTEM parameters within normal limits (Figure 3). Following consultation with haematology, no additional transfusions or coagulation factors were administered due to the patient’s high thrombotic risk.

**Table 3:** Intraoperative laboratory results obtained concurrently with second ROTEM analysis ROTEM: rotational thromboelastometry; * indicates values outside of the normal reference range

Parameter	Normal range	Results
White blood cells (K/μL)	4.5-10.5	2.5*
Neutrophils (%)	40.0-70.0	25.0*
Lymphocytes (%)	20.0-45.0	60.5*
Monocytes (%)	2.0-12.0	14.1*
Eosinophils (%)	0.0-6.0	0.3
Basophils (%)	0.0-2.0	0.1
Neutrophils (K/μL)	1.8-7.0	0.6*
Lymphocytes (K/μL)	1.2-3.8	1.5
Monocytes (K/μL)	0.2-1.0	0.4
Eosinophils (K/μL)	0.0-0.6	0.0
Basophils (K/μL)	0.0-0.2	0.0
Red blood cells (M/μL)	4.5-6.00	3.1*
Haemoglobin (g/dL)	14.0-17.5	9.3*
Haematocrit (%)	42.0-52.0	26.4*
Mean corpuscular volume (MCV, fL)	79.0-98.0	83
Mean corpuscular haemoglobin (MCH, pg)	26.0-32.0	29.5
Mean corpuscular haemoglobin concentration (MCHC, g/dL)	32.0-36.0	35.6
Red blood cell distribution width-coefficient of variation (RDW-CV, %)	11.5-14.9	14.3
Platelets (K/μL)	140-440	52*
Mean platelet volume (MPV, fL)	7.0-10.5	7.6
Plateletcrit (%)	0.140-0.420	0.042*
Platelet distribution width-coefficient of variation (PDW, %)	12.0-18.0	16.6
Prothrombin time (PT, sec)	11.0-15.0	10.1*
International normalised ratio (INR)	-	0.94
Activated partial thromboplastin time (APTT, sec)	26.0-40.0	21.4*
Fibrinogen (g/L)	2.0-4.0	3.8

**Table 4:** Postoperative arterial blood gas analysis Hgb: haemoglobin; Hct: haematocrit; HCO_3_: bicarbonate; SO_2_: oxygen saturation; pCO_2_: partial pressure of carbon dioxide; pO_2_: partial pressure of oxygen Nasal cannula, 2 L/min

Parameter	Result
pH	7.39
pCO_2_	39
pO_2_	78
Na^+^	135
K^+^	3.7
Ca^++^	11.0
Glucose	87
Lactate	2.4
Hgb	9
Hct	28
HCO_3_	23.6
Base excess	-1.4
SO_2_	99%

The patient was then transferred to the Post-Anaesthesia Care Unit (PACU), where she was monitored for 24 hours. Prophylactic anticoagulation with enoxaparin 40 mg subcutaneously once daily was initiated on the same day. Her vital signs remained stable, and her laboratory tests performed the following day showed a haematocrit of 30.5%, haemoglobin of 10 g/dl, platelet count of 46 x 103/mcl, and white blood cell count of 2.5 x 103/mcl (Table 5). Consequently, no further transfusions were deemed necessary.

**Table 5:** Laboratory findings on the first postoperative day * indicates values outside of the normal reference range

Parameter	Normal range	Results
White blood cells (K/μL)	4.5-10.5	2.5*
Neutrophils (%)	40.0-70.0	32.1*
Lymphocytes (%)	20.0-45.0	54.1*
Monocytes (%)	2.0-12.0	13.4*
Eosinophils (%)	0.0-6.0	0.2
Basophils (%)	0.0-2.0	0.2
Neutrophils (K/μL)	1.8-7.0	0.8*
Lymphocytes (K/μL)	1.2-3.8	1.4
Monocytes (K/μL)	0.2-1.0	0.3
Eosinophils (K/μL)	0.0-0.6	0.0
Basophils (K/μL)	0.0-0.2	0.0
Red blood cells (M/μL)	4.5-6.00	3.4*
Haemoglobin (g/dL)	14.0-17.5	10.0*
Haematocrit (%)	42.0-52.0	30.5*
Mean corpuscular volume (MCV, fL)	79.0-98.0	84
Mean corpuscular haemoglobin (MCH, pg)	26.0-32.0	29.7
Mean corpuscular haemoglobin concentration (MCHC, g/dL)	32.0-36.0	35.6
Red blood cell distribution width-coefficient of variation (RDW-CV, %)	11.5-14.9	14.4
Platelets (K/μL)	140-440	46*
Mean platelet volume (MPV, fL)	7.0-10.5	8.0
Plateletcrit (%)	0.140-0.420	0.036*
Platelet distribution width-coefficient of variation (PDW, %)	12.0-18.0	17.0
Potassium (K, mmol/L)	3.5-5.1	3.9
Sodium (Na, mmol/L)	136-145	141
Alanine transaminase (ALT, U/L)	<41	6
Aspartate transaminase (AST, U/L)	<37	16
Urea (mg/dL)	6-22	20
Glucose (mg/dL)	65-110	93
Creatinine (mg/dL)	0.6-1.2	0.46*
Gamma-glutamyl transferase (GGT, U/L)	11-49	14
Uric acid (mg/dL)	<5.7	5.2
Total bilirubin (mg/dL)	0.2-1.2	0.42
Total proteins (g/dL)	6.4-8.3	5.4*
Albumin (g/dL)	3.5-5.0	3.2*
C-reactive protein-high sensitive (mg/dL)	<0.4	5.53*
Lactate dehydrogenase (LDH, U/L)	135-225	268*
Prothrombin time (PT, sec)	11.0-15.0	11.6
International normalised ratio (INR)	-	1.09
Activated partial thromboplastin time (APTT, sec)	26.0-40.0	36.2
Fibrinogen (g/L)	2.0-4.0	5.3

## Discussion

This case highlights several important considerations in the anaesthetic management of parturients with PNH and AA. Such cases are rare and typically limited to AA patients with a PNH clone, a group that appears to experience better pregnancy outcomes [5]. Anaesthetic management should take into account complications related to both conditions. To the best of our knowledge, this is the first reported case in literature that describes in detail the anaesthetic management of a parturient with both PNH and such severe AA undergoing caesarean section.

From an anaesthetic perspective, the primary concern in PNH is preventing complement activation, which may be triggered by physiological or pharmacological stress, such as pain, trauma, surgery, or infection. Perioperative inflammation and acidosis may further exacerbate this process, underlining the importance of maintaining homeostasis and avoiding hypoxaemia, hypercapnia, hypoperfusion, hypothermia, and dehydration [8-12]. Severe thrombocytopaenia in AA-PNH patients raises the risk of perioperative bleeding. Perioperative administration of eculizumab is essential to minimise haemolysis, while there are case reports that describe increasing the dose or frequency of eculizumab perioperatively to ensure adequate complement inhibition [10,12]. In our case, these principles guided management, including vigilant haemodynamic monitoring with invasive arterial access, the continuation of eculizumab therapy and minimisation of perioperative fasting. Targeted perioperative management is especially critical in PNH obstetric patients, where the physiologic demands of pregnancy and delivery further increase the risk of haemolysis and maternal-fetal complications.

Both regional and general anaesthesia have been successfully used in PNH patients. Regional anaesthesia is preferred for caesarean section, since it avoids airway manipulation and is associated with better maternal and neonatal outcomes. It also provides excellent postoperative analgaesia, which is crucial in these patients to avoid complement activation. An absolute platelet threshold for performing neuraxial procedures is yet to be established. However, in patients with PNH or AA, thrombocytopaenia can complicate or even preclude the use of regional anaesthesia [8-13]. General anaesthesia can be performed regardless of any coagulation disorders and is useful in emergency situations. Nonetheless, it involves airway instrumentation and transplacental transfer of anaesthetic agents, which may affect the neonate. If general anaesthesia is performed, it is important to ensure adequate anaesthetic depth and effective intra- and postoperative analgaesia to avoid stress-induced complement activation [8-12]. Among anaesthetic agents, propofol does not seem to affect complement activity, while volatile agents may be able to decrease complement levels, even though this potential benefit requires further investigation [9-11,14]. Although prolonged or repeated exposure to N₂O has been associated with potential myelodepressive effects, there are reported cases of its safe administration in patients with PNH [9-11]. In our case, the platelet count was critically low despite transfusion, justifying the choice of general anaesthesia. The selected technique proved safe, as induction and maintenance were uneventful, and multimodal analgaesia successfully limited physiological stress postoperatively. Ultimately, anaesthetic technique in AA-PNH patients should be personalised and guided by individual risk factors, maternal and fetal safety, coagulation status, and the need to minimise complement activation.

There is currently very limited literature on the use of ROTEM in PNH or AA. While ROTEM is a valuable tool for assessing coagulation status in various clinical scenarios, its specific application in PNH or AA has not been extensively studied. To our knowledge, there is only one study that assessed clot formation using ROTEM in PNH patients. ROTEM analysis in these patients revealed slower formation of smaller clots, which correlated with their platelet count [15]. In our patient, serial ROTEM assessments mirrored these observations. The first ROTEM, obtained after the patient had received three preoperative platelet units, showed prolonged EXTEM clot formation time and reduced clot amplitudes, indicating delayed and weak clot formation. This prompted the transfusion of an additional platelet unit. A subsequent ROTEM showed improvement in EXTEM parameters. However, FIBTEM results revealed reduced early fibrin-based clot firmness, indicating impaired fibrinogen contribution to clot formation. Following our institutional protocol, 1 g of tranexamic acid was administered. Tranexamic acid is an antifibrinolytic agent that preserves existing fibrin clots from premature lysis, supporting haemostasis even when fibrin-based clot formation is impaired. Additional pro-coagulant therapy was intentionally withheld due to the patient’s high thrombotic risk. Further research is needed to explore the potential role of ROTEM in the evaluation and management of patients with these haematologic disorders, particularly in emergency or situations requiring rapid interventions.

Beyond anaesthetic technique and haemostasis, PNH in pregnancy carries additional risks. Preeclampsia and haemolysis, elevated liver enzyme levels and low platelet (HELLP) syndrome occur at higher rates in PNH compared to healthy parturients; therefore, PNH patients should be closely monitored [6,16]. Interestingly, preeclampsia alone also enhances complement activation, potentially worsening the disease [17]. Thrombosis remains the leading cause of morbidity and mortality in these patients, and prophylactic anticoagulation is indicated even in the presence of low platelet counts, particularly during the high-risk postpartum period [1,6,7]. Obstetric haemorrhage also remains a concern, potentially linked not only to anticoagulation, but also to PNH-related smooth muscle dystonia [16]. Breakthrough haemolysis frequently occurs during pregnancy, even in patients receiving eculizumab, possibly due to increased complement activation. In such cases, the dose of eculizumab should either be increased or the dose interval should be shortened [7,12,16]. In our patient, prophylaxis was initiated on the day of delivery with a platelet count of 52 × 10³/mcl, balancing bleeding and thrombotic risks. Given the complex interplay between obstetric complications, thrombosis, and haemolysis in these patients, individualised interventions are essential to optimise maternal and fetal outcomes.

Another important consideration is the potential effect of the treatments on the fetus. Limited data are available regarding the impact of eculizumab on the fetus, including permeability across the placenta and excretion in breast milk. Existing studies suggest that therapeutic doses of eculizumab do not impair the neonatal complement system and may be safe to use during pregnancy [7,18]. However, there are studies detecting eculizumab in cord blood samples, indicating that it can cross the placenta [19,20]. More research is needed to study its use in this special setting.

Finally, the multi-system complications of PNH underscore the need for thorough preoperative evaluation. Due to the high level of haemolysis in these patients, free haemoglobin, which is normally cleared by haptoglobin, binds to nitric oxide, reducing its bioavailability. Therefore, decreased nitric oxide concentration may be accountable for arterial and pulmonary hypertension, kidney damage, headaches, increased platelet activation, and oesophageal contractility disorders [1,2]. Preoperative workup should therefore include full blood count, coagulation, and chemistry panels (including LDH), and consideration of ECG, echocardiography, and pro-brain natriuretic peptide (proBNP) to assess cardiopulmonary involvement [12]. Reviewing prior transfusion history is essential, as repeated transfusions increase the risk of HLA sensitisation [13]. This is particularly important in PNH patients, where transfusion reactions may trigger complement activation and result in breakthrough haemolysis. Additionally, immunocompromised status should be carefully considered, with antibiotic prophylaxis given before incision and strict precautions applied during invasive procedures [9,13]. In our case, a comprehensive preoperative evaluation was carried out one week prior to caesarean section, involving close coordination with the obstetric and haematology teams, alongside early notification of the blood bank to ensure timely access to blood products. Such meticulous multidisciplinary planning is essential to minimise perioperative risks for both mother and fetus.

## Conclusions

Pregnancies complicated by PNH-AA require a multidisciplinary team approach. Anaesthetic management of these patients requires coordinated communication with a variety of other specialists, like haematologists, obstetricians, and neonatologists. An anaesthetic plan should be formulated in advance, incorporating considerations such as the degree of haemolysis, severity of cytopaenias, and the risk of bleeding or infection. Perioperative management should be carefully implemented, taking into account multiple factors such as analgaesic and anticoagulant use, in order to balance the risks of bleeding and thrombosis. This collaborative approach should be implemented throughout the entire perioperative period to minimise complications and ensure optimal maternal and neonatal outcomes.

In this context, ROTEM may offer useful, real-time information to guide transfusion and coagulation management and could potentially assist in balancing the dual risks of bleeding and thrombosis. However, its role in the perioperative management of patients with bone marrow failure syndromes remains to be established. Given the limited literature, additional case reports and clinical studies are necessary to guide future anaesthetic care in this high-risk population.
